# Impact of inherent aspects of body image, eating behavior and perceived health competence on quality of life of university students

**DOI:** 10.1371/journal.pone.0199480

**Published:** 2018-06-22

**Authors:** Wanderson Roberto da Silva, Juliana Alvares Duarte Bonini Campos, João Marôco

**Affiliations:** 1 Department of Food and Nutrition of School of Pharmaceutical Sciences, São Paulo State University (UNESP), Araraquara, São Paulo, Brazil; 2 William James Center for Research (WJCR), University Institute of Psychological, Social, and Life Sciences (ISPA), Lisbon, Portugal; Hong Kong Polytechnic University, HONG KONG

## Abstract

The aim of this study was to assess the impact of inherent aspects of body image, eating behavior and perceived health competence on quality of life of university students. Participants completed the instruments Body Shape Questionnaire (reduced version, BSQ-8B), Male Body Dissatisfaction Scale (reduced version, MBDS-R), Three-Factor Eating Questionnaire (reduced version, TFEQ-18), Perceived Health Competence Scale (bifactorial version, PHCS-B), World Health Organization Quality of Life Questionnaire-Short Form (WHOQoL-bref) and a questionnaire for characterization of sample. Psychometric properties of instruments were previously evaluated by confirmatory factor analysis. A hypothetical model for each sex was developed and tested. In both model surveys the aspects of the body image (BSQ-8B: body shape concern; MBDS-R: musculature and general body appearance), of eating behavior (TFEQ-18: cognitive restriction, emotional eating, and uncontrolled eating) and of the perceived health competence (PHCS-B: expectations of achieving the desired health results and competence in health behaviors) were used as direct predictors on quality of life (WHOQoL-bref). The variables age, medication use for body change, food supplement use for body change, and body mass index (BMI) were inserted in the aspects of the body image. The variables course shift, initial expectation regarding the course, self-reported performance in the course, concomitant work activities to studies, and economic class were inserted into the quality of life. The model surveys were evaluated using structural equation modeling. A level of significance of 5% was used. A total of 2,198 university students (female = 63.5%), including 1,151 Brazilians and 1,047 Portuguese, participated of study (locally representative samples). The average age of women was 20.8 ± 2.4 years and of men was 21.3 ± 3.3 years. The psychometric properties of the instruments were adequate, except for the PHCS, which was adjusted for each sex. The models presented variance explained of 54% and 49% for women and men, respectively. In both sexes, the students’ perceived health competence and academic variables contributed significantly to their quality of life, and age, BMI, and medication and supplement use were significant factors relating to how a student views his or her body image. Women's quality of life was associated with body shape concern and emotional eating aspects. Men's quality of life was associated with general body appearance and cognitive restriction aspects. These results can be used to create and implement educational programs to improve quality of life of university students.

## Introduction

The quality of life of individuals is a topic of concern of specialists from several fields; however, there is no consensus in the literature about what this concept means, which may be a result of its complexity and multidimensionality. Some research has used the term based on the meaning of it proposed by the World Health Organization (WHO) that sees it as the individual’s perceptions about his or her goals, expectations, patterns and concerns, and his or her position in life in the context of culture and systems of values in which they live [1]. Fayers and Machin [2] report that to measure the quality of life, it is important to understand the magnitude of this concept, which involves mainly psychological, physical and social domains. The evaluation of a person’s quality of life may be done several ways; however, the use of psychometric instruments has been a research method commonly adopted by researchers and clinicians. General instruments that evaluate the individual’s quality of life without considering a specific domain in an individual’s experience are available in the literature and aid in this research. The quality of life group of the WHO (WHOQOL) developed the World Health Organization Quality of Life Questionnaire (WHOQoL) with the collaboration of researchers from several countries. This instrument was created to evaluate the main domains related to the quality of life of people in different cultures. The final version of the instrument was presented with 100 items. However, to aid in broad epidemiological studies, a shorter version (WHOQoL-bref) was proposed by the WHOQOL group [1], and this has been commonly used by researchers.

As well as the importance of measuring the quality of life, Fayers and Machin [2] emphasize the need to create theoretical models that seek to evaluate which aspects of individuals' lives impact their quality of life. However, there are multiple aspects that influence an individual’s quality of life and, therefore, the choice of specific domains should be based on theoretical premises supported by previous studies and the purpose of the research. In the last few years, research related to body image, eating behavior and perceived health competence have highlighted that these concepts play a role in people's quality of life. Sanftner [3] identified a significant impact of perceptions about body image and eating behavior on the quality of life of Americans. Rueda and Perez-Garcia [4] observed the significant influence of perceived health competence in the quality of life of Spaniards. Thus, considering the relationship between these concepts, the it is important to build a theoretical model to take into account these concepts and how they impact on individual’s quality of life, once the literature has provided information that supports premises in the model [3–5].

Aspects relating to body are described in the literature as body image. Body image is a concept defined by Cash and Smolak [6] as the mental representation that individual makes in relation to his or her own body. Most body image research reports the multidimensionality of this concept, which is usually evaluated by looking at different aspects that compose the perceptual and/or attitudinal dimensions of body image. The attitudinal dimension is the most evaluated in the literature due to the wide availability of psychometric instruments that are used to evaluate beliefs, emotions, concerns, behaviors, and satisfaction/dissatisfaction of individuals with their own bodies. Body shape concern and body dissatisfaction are examples of beliefs that are commonly measured to evaluate the attitudinal dimensions of body image. Cox et al. [7] warn that individuals with greater concern/dissatisfaction with the body are more vulnerable to the development of eating disorders and body dysmorphia. These disorders may have significant impact on the lives of individuals. Concerned about these issues, Cooper et al. [8] developed the Body Shape Questionnaire (BSQ) to assess women's body shape concerns, and Ochner, Gray and Brickner [9] developed the Male Body Dissatisfaction Scale (MBDS) to assess men’s body dissatisfaction. These instruments were proposed to consider the differences between women and men regarding their perception of body image.

Cash and Smolak [10] point out that the concern with body is a common characteristic of both sexes. However, the authors warn that there are significant differences in bodily perceptions of women and men. Women see body fitness in the context of fat loss and the increase/definition of lower body parts, while men value muscle and the increase/definition of the upper body [11]. Thus, instruments that provide an adequate picture of the concerns of each sex regarding body image should be developed carefully. Thus, the construction of theoretical models that include the body image should be planned considering these peculiarities. In addition, the literature has also pointed out that body image concept is directly related to age [12], body weight [13,14] and the use of medication and supplements that promise body changes [15,16]. Therefore, these are important variables to keep in mind when developing a study.

Despite being a widely evaluated issue, there is no consensus in the literature about how to define or study eating behavior. In general, it can be said that eating behavior is a set of cognitions and affections regarding food that are strongly related to psychological and socio-cultural issues. Considering that eating behavior mainly involves the experiences of individuals with food, psychometric instruments have been recommended to understand "how" and "why" people have certain eating behaviors. Among these instruments, the most cited may be the Three-Factor Eating Questionnaire (TFEQ) [17]. The TFEQ is used to measure inherent aspects of eating behavior, and its reduced version of 18 items (TFEQ-18) [18] has been recommended for evaluation of the cognitive restriction, emotional eating, and uncontrolled eating that can have an impact on people's lives.

Considering that quality of life may be associated with health, the evaluation of individuals' perceptions regarding health management may be interesting [19,20]. According to Smith, Wallston, and Smith [21] self-efficacy or perceived competence is an important construction in predicting how individuals take care of their own health. The Perceived Health Competence Scale (PHCS) [21] was proposed to evaluate individuals' self-efficacy beliefs regarding health behaviors and results, and has been used in clinical and non-clinical samples. The significant relation between perceived health competence evaluated through PHCS, and quality of life, has been presented in some studies with clinical samples [4,19,20,22]. However, the relation of this concept with the quality of life in non-clinical samples is not often explored and, therefore, this investigation is of interest, since it may aid in the identification of individuals who need encouragement and additional preventive support for self-care.

Besides the inherent aspects of body image, eating behavior, and perceived health competence, some researchers have pointed out that social, demographic and economic factors may influence on quality of life. Shareef et al. [23] go further and report that when evaluating the quality of life in a non-clinical population, such as young university students, specific factors regarding the environment can also directly influence on quality of life. These authors emphasize the importance of evaluating the quality of life and the interference of different aspects in the university population, once these individuals are in sudden life transitions and enter the autonomy phase of adulthood. Thus the university itself may act as a stressful environment. Several national and international studies seek to evaluate the quality of life of university students. However, as pointed out by Fayers and Machin [2] it is necessary to construct a theoretical model to broadly identify direct influences on the lives of these students. It is known that the construction of a single theoretical model that may attend to different samples/populations would be useful for studying these issues. The scientific community has shown a growing interest in studies that include samples from different countries in order to obtain stronger evidence and conclusions on these topics. It is important to highlight that the variables included in this study were not evaluated simultaneously in previous studies and they were used as independent predictors, since there is no rationale explaining to use mediator paths. In this context, this study was aimed to assess the impact of inherent aspects of body image, eating behavior, and perceived health competence on the quality of life of university students.

## Methods

### Study and sample design

The is a cross-sectional study with non-probabilistic sampling designed by convenience. To calculate the sample size, one of the recommendations of Hair, Black, Babin, and Anderson [24] was considered, that the study have at least 5 respondents per parameter evaluated in the model. Considering that in this study the theoretical model to be tested includes aspects related to body image, it was decided to create separate models for women and men seeking to respect the difference for each sex. Thus, the calculation of the sample size was performed for each sex. The complete model tested for women presented 130 parameters (instruments/variables: BSQ-8B + TFEQ–18 + PHCS-B + sociodemographic + WHOQoL-bref) resulting in a minimum sample size of 650 subjects. Then, the complete model tested for men presented 128 parameters (instruments/variables: MBDS-R + TFEQ–18 + PHCS-B + sociodemographic + WHOQoL-bref) resulting in a minimum sample size of 640 subjects. It is important to highlight that despite the calculations have been performed to attend to the final structural models, the sample sizes also were adequate to evaluate the quality of the adjustment of factorial models of each instrument to the data.

### Participants

Seeking to enlarge the application of the theoretical model tested in this study, students from different Portuguese speaking countries were included. The data used in the present study were obtained from a larger dataset, which we collected previously (i.e. primary data), composed of Brazilian, Portuguese and Mozambican university students (regionally representative samples). However, the data from Mozambique were not used because the sample size was insufficient to carry out the analyzes. Thus, this study had the voluntary participation of Brazilian and Portuguese university students of both sexes. In Brazil, we were invited to join students of the School of Pharmaceutical Sciences (FCF), School of Sciences and Letters (FCL) and Institute of Chemistry (IQ) from the São Paulo State University (UNESP, campus of Araraquara). In Portugal, were invited to join the students of the University Institute of Psychological Sciences, Social and Life (ISPA), Health Sciences Institute Egas Moniz (ISCSEM), School of Pharmacy of University of Coimbra (FFUC), Nursing School of Lisbon (ESEL) and High Institute of Engineering from Porto (ISEP). The inclusion criteria adopted were: to be between 18 and 35 years old, to be duly enrolled in an undergraduate course of the above-mentioned institutions, and for women to not be in the gestation period. It should be clarified that age restriction was adopted because some studies reported that the perception of people regarding to body image [6,12], eating behavior [25,26] and health competence [27,28] may differ according to an individual's age, and considering that the university population consists mostly of young adults, we restricted the age to between 18 and 35 years.

### Procedures

All higher education institutions were informed about the research and approved data collection in the classroom with the presence of a teacher responsible for discipline at the time of collection. After the teachers' knowledge and agreement, a schedule with each class was created, and the students were informed and invited to answer the instruments with an average duration of approximately 20 minutes. Only eligible students (in accordance with the inclusion criteria) and those who agreed to sign the Free and Informed Consent Term were included in the study sample and no student refused to complete the questionnaires. Additionally, it is important to report that the randomization of the instruments was performed in order to minimize possible biases because of a unique sequence of questionnaires. This study followed the ethical precepts dictated by the Resolution 466/2012 of the National Health Council and was approved in Brazil by the Human Research Ethics Committee of the School of Pharmaceutical Sciences of São Paulo State University (C.A.A.E. 29896214.0.0000.5426) and in Portugal by the Nursing School of Lisbon (protocol 1413).

### Sample characterization and study variables

Information regarding age, sex, housing, presence of work activity concomitant to studies, area, year and period of the course of study, initial expectations regarding the course of study, thoughts about giving up the course of study, self-reported performance in the course, frequency of medication consumption due to studies, and frequency of medication consumption and food supplements for body change were collected. The economic class of participants was also obtained using Brazilian Economic Classification Criteria [29] and in Portugal, the family's average monthly income was obtained in minimum wages considering the values presented by the government agency (www.ine.pt). It should be clarified that the difference in the methodology used to determine the economic class in the two countries was based on the different recommendations of each country to evaluate this variable.

The body weight and height self-reported by the students were used to calculate the body mass index (BMI). The nutritional status of the participants was obtained following the recommendations of the World Health Organization [30,31]. It should be clarified that the use of self-reported weight and height is commonly observed in the literature in epidemiological studies. Besides, we conducted a pilot study with 356 students and verified that the degree of agreement between "self-reported weight and height" and "measured weight and height" was high [Intraclass Correlation Coefficient: weight = .98 (95% CI .97-.98); height = .97 (95% CI .96-.97), which supported the use of self-reported measures.

The aspects of the body image (body shape concern, musculature, and general body appearance), of eating behavior (cognitive restriction, emotional eating, and uncontrolled eating) and of perceived health competence (expectations of achieving the desired health results, and competence in health behaviors) were measured by psychometric instruments as well the quality of life concept. The instruments are described below.

### Instruments

In view of the recommendations already mentioned in the introduction of this study regarding the evaluation of body image in women and men, it was decided to use different instruments to evaluate this concept in each sex.

#### Body Shape Questionnaire (BSQ)

The BSQ was originally proposed by Cooper et al. [8] in the English language to measured women’ body shape concerns. This instrument consisted of 34 items with a 6-point Likert type response scale grouped into a single domain named “Body Shape Concern”. Evans and Dolan [32] after verifying the redundancy of the items in the questionnaire, suggested the use of reduced versions respecting the original theoretical proposal of one domain. Later, Da Silva, Dias, Marôco and Campos [33] tested all the reduced versions suggested by Evans and Dolan and revealed that the B version of 8 items (items = 5, 11, 15, 20, 21, 22, 25, 28) was the most efficient with adequate validity and reliability for a sample of Brazilian university students. Silva, Costa, Pimenta, Marôco and Campos [34] also found that reduced version 8B was the most adequate in a sample of Brazilian and Portuguese students. The authors also presented a Portuguese version reconciled for Brazil and Portugal. Thus, in this study, the reduced versions of BSQ (BSQ-8B) was used to measure women’ body shape concern.

#### Male Body Dissatisfaction Scale (MBDS)

The MBDS was originally proposed by Ochner et al. [9] in the English language with 25 items (13 formulated in the opposite direction; items = 4, 5, 6, 7, 9, 10, 12, 13, 17, 22, 24, 25) to assess the body dissatisfaction of men considering Musculature (items = 4, 6, 7,9, 12, 13, 16, 24), Definition (items = 1, 3, 10, 15, 17, 18, 20, 22, 25), and Relative Positioning/External Evaluation (items = 2, 5, 8, 11, 14, 19, 21, 23) domains. The items of instrument were developed with two sets of answers to be completed, one referring to the importance assigned to the item (ranging from 1 to 10) and another one related to the agreement/frequency with the item (5-point Likert-type scale). The weight of each item is obtained by dividing the value assigned to the importance by 10, and then multiplying this value by the participant's response to the Likert-type scale of that same item (each item's score ranging from .1 to 5.0 points). The Portuguese version of the MBDS was presented by Carvalho et al. [35] and was the one used in this study. Da Silva, Marôco, Ochner and Campos [36] evaluated the construct validity of the MBDS and verified that the original version did not fit the sample of Brazilian and Portuguese university students. These authors also reported the need to perform a theoretical review of the contents of each item and allocation of the domains. After this review, Da Silva et al. [36] proposed a reduced version of 12 items (5 formulated in the opposite direction; items = 4, 6, 9, 12, 16) distributed in 2 domains (Musculature: items = 4, 6, 9, 12, 16; General Body Appearance: items = 1, 2, 8, 15, 19, 21, 23), which presented adequate validity and reliability for the sample of students. Thus, in this study, the reduced version of MBDS (MBDS-R) was used to measure men’ body image aspects.

#### Three-Factor Eating Questionnaire (TFEQ)

The TFEQ was originally proposed by Stunkard and Messick [17] in the English language to assess eating behavior of women and men. This instrument consisted of 51 items (7 formulated in the opposite direction; items = 10, 16, 21, 25, 30, 31, 47) with response scales dichotomous or Likert type of 4 and 6 points, and 3 domains (Cognitive Restriction: items = 4, 6, 10, 14, 18, 21, 23, 28, 30, 32, 33, 35, 37, 38, 40, 42, 43, 44, 46, 48, 50; Disinhibition: items = 1, 2, 7, 9, 11, 13, 15, 16, 20, 25, 27, 31, 36, 45, 49, 51; Hunger: items = 3, 5, 8, 12, 17, 19, 22, 24, 26, 29, 34, 39, 41, 47). Karlsson, Persson, Sjöström and Sullivan [18] evaluated the TFEQ-51 and identified the need for restructuring of the instrument that was composed of 18 items (TFEQ-18) allocated in 3 different domains from the original proposal (Cognitive Restriction = 6, 28, 33, 43, 48, 50; Emotional Eating = 9, 20, 27; Uncontrolled Eating = 1, 15, 19, 22, 24, 26, 34, 39, 49). This reduced version has been used in some studies and considered suitable for different samples. In this way, for the present study the inherent aspects of eating behavior were measured in women and men using the TFEQ-18. The Portuguese version [37] of the instrument reconciled between Brazil and Portugal was used for the present study.

#### Perceived Health Competence Scale (PHCS)

The PHCS was originally proposed by Simith, Wallston and Smith [21] in the English language to assess the individuals’ perceived heath competence. This instrument was developed for use in women and men and consisted of 8 items (4 formulated in the opposite direction; items = 1, 2, 6, 7) with a 5-point Likert type response scale grouped into a single domain named “Perceived Health Competence”. The scale was translated into Portuguese and reconciled between Brazil, Portugal, and Mozambique by Silva, Pimenta, Marôco, Maloa and Campos [28]. The authors of the reconciled Portuguese version evaluated the psychometric properties of different PHCS models, including the original, and verified that a bifactorial version (Expectations of Achieving the Desired Outcomes in Health: items = 1, 2, 6, 7; Competence in Health Behaviors: items = 3, 4, 5, 8) was the most parsimonious for the study sample. Therefore, the inherent aspects of perceived health competence were measured using this proposal of PHCS (PHCS-B) in women and men.

#### World Health Organization Quality of Life Questionnaire-Short Form (WHOQoL)

The WHOQoL was originally developed by WHOQOL group in collaboration with 15 international centers[1] to evaluate the quality of life of individuals of both sexes. The instrument was initially composed by 100 questions and 6 domains (Physical; Psychological; Level of Independence; Social Relationships; Environment; Spirituality/Religion/Personal Beliefs); however, a reduced version (WHOQOL-Bref) was proposed to be used in epidemiological contexts. The WHOQOL-Bref consists of 26 items (3 formulated in the opposite direction; items = 3, 4, 26) with a 5-point Likert type response scale grouped into 4 domains (Physical: items 3, 4, 10, 15, 16, 17, 18; Psychological: items = 5, 6, 7, 11, 19, 26; Social Relations: items = 20, 21, 22; Environment: items = 8, 9, 12, 13, 14, 23, 24, 25). It should be clarified that the first two items of the questionnaire (1 = general quality of life and 2 = general health) are complementary and are not inserted in the factorial model of the instrument. The Portuguese version of the WHOQoL-bref was presented by Fleck et al. [38]. Silva, Bonafé, Marôco, Maloa and Campos [39] evaluated the psychometric properties of the WHOQoL-bref in different samples and pointed out that a structure of 20 items (1 formulated in the opposite direction; item = 26), 4 domains of first order (Physical: items = 10, 16, 17, 18; Psychological: items = 5, 6, 7, 11, 19, 26; Social Relations: items = 20, 21, 22; Environment: 9, 12, 13, 14, 23, 24, 25), and 1 domain of second order (Quality of life) presented adequate validity and reliability for the samples of Brazilian and Portuguese university students. Therefore, this refined version of WHOQoL-bref was used in the present study to measure quality of life in both women and men.

#### Instruments’ psychometric analysis

Before the elaboration of the structural models, the psychometric properties of each instrument were evaluated. Only the instruments fully completed were used, that is, with all the items properly filled out by the student. The psychometric properties of each instrument for the study sample were evaluated. This evaluation was performed separately for each sex and then for each country (Brazil and Portugal) to ensure the adequacy of the adjustment of the instruments to the samples.

The factorial validity was evaluated through confirmatory factorial analysis (CFA) using the Weighted Least Squares Mean and Variability Adjusted (WLSMV) method in the polychoric correlation matrix for the instruments with categorical responses and the Maximum Likelihood method in Pearson matrix for the MBDS-R, because it presents continuous answers. The indices chi-square ratio by degrees of freedom (χ^2^/*df*), Root Mean Square Error of Approximation (RMSEA) with confidence interval of 90% (CI 90%), Comparative Fit Index (CFI) and Tucker-Lewis Index (TLI) were used to verify the quality of the instrument models adjustment [40]. Values of χ^2^/*df* ≤ 5.0, RMSEA ≤ .10, CFI ≥ .90, and TLI ≥ .90 were considered acceptable quality indicators of adjustment [40]. The factorial weight (λ) of each item was also evaluated and values higher than .40 were considered adequate [40]. When the fit was not adequate, the modification indexes higher than 11 (*p* < .001), calculated from the Lagrange Multipliers (LM), were analyzed. These analyses were performed in the software MPLUS version 7.2 (Muthén and Muthén, Los Angeles, USA, 2014).

The convergent and discriminant validities were evaluated from the calculation of the average variance extracted (AVE) and the coefficient of determination between domains (*r*^2^), respectively [41]. Values of AVE *≥* .50 and *r*^2^_ij_ < AVE_i_ and AVE_j_ were considered indicators of convergent and discriminant validity, respectively [40].

The reliability was evaluated by the composite reliability (CR) and by the internal consistency. The CR was evaluated following the proposal of Fornell and Larcker [41] and the internal consistency was evaluated from the alpha coefficient (*α*) of Cronbach. CR and α values greater than .70 indicated adequate reliability [40].

Table 1 presents the indicators for evaluation of the psychometric properties of each instrument used in this study. For the female sample, it is noted that the BSQ-8B, TFEQ-18, and WHOQoL-bref presented adequate adjustment without the need of modifications. Yet the PHCS-B presented acceptable psychometric qualities only after the insertion of two correlations between item errors. For the male sample, it is noted that MBDS-R, TFEQ-18, and WHOQoL-bref were adequate without modification. On the other hand, two items of PHCS-B were excluded for scale adjustment to the male population. In both female and male samples, a lack of discriminant validity in the PHCS-B domains, a low convergent validity in the psychological and environmental domains of the WHOQoL-bref, and internal consistency in the limit of acceptable for the social relations domain of WHOQoL-bref was observed and these results were also verified in other works [28,42].

**Table 1:** Indicators for evaluation of the psychometric properties of the instruments separated for each sex and country.

Instrument	Country	n	χ^2^/*df*	RMSEA [CI 90%]	CFI	TLI	λ	EI	e	*r*^2^	AVE	CR	α
Female													
BSQ-8B	BR/PT	1,396	9.97	.08 [.07-.09]	.98	.98	.67-.82	-	-	-	.57	.91	.88
BSQ-8B	BR	722	6.69	.09 [.07-.10]	.98	.97	.67-.80	-	-	-	.56	.91	.88
BSQ-8B	PT	674	4.70	.07 [.05-.09]	.99	.98	.67-.83	-	-	-	.58	.92	.88
PHCS-B	BR/PT	1,396	18.98	.11 [.10-.12]	.97	.96	.54-.86	-	-	.72	.55-.59	.82-.85	.77-.80
PHCS-B (fitted)	BR/PT	1,396	10.79	.08 [.07-.09]	.99	.98	.54-.84	-	3–4, 4–5	.77	.54-.55	.82-.82	.77-.80
PHCS-B	BR	722	9.97	.11 [.10-.13]	.97	.96	.58-.85	-	-	.76	.54-.55	.82-.83	.77-.78
PHCS-B (fitted)	BR	722	6.12	.08 [.07-.10]	.98	.97	.58-.82	-	3–4, 4–5	.83	.50-.54	.80-.82	.77-.78
PHCS-B	PT	674	10.42	.12 [.10-.13]	.97	.96	.51-.86	-	-	.70	.57-.62	.83-.87	.76-.81
PHCS-B (fitted)	PT	674	6.80	.09 [.08-.10]	.99	.98	.51-.87	-	3–4, 4–5	.74	.57-.58	.83-.84	.76-.81
TFEQ-18	BR/PT	1,396	4.34	.05 [.04-.05]	.95	.95	.57-.87	-	-	.00*-.33	.45-.69	.87-.88	.71-.77
TFEQ-18	BR	722	3.19	.05 [.05-.06]	.94	.93	.57-.86	-	-	.00*-.27	.46-.69	.86-.88	.69-.78
TFEQ-18	PT	674	2.13	.04 [.03-.05]	.96	.96	.53-.88	-	-	.00*-.34	.44-.69	.86-.87	.70-.76
WHOQoL-bref	BR/PT	1,396	7.99	.07 [.06-.08]	.94	.93	.45-.90	-	-	.26-.60	.32-.58	.76-.84	.65-.77
WHOQoL-bref	BR	722	4.14	.07 [.06-.07]	.94	.93	.41-.92	-	-	.27-.59	.30-.57	.74-.84	.67-.77
WHOQoL-bref	PT	674	5.24	.08 [.07-.08]	.92	.90	.40-.94	-	-	.23-.57	.37-.55	.77-.84	.63-.78
Male													
MBDS-R	BR/PT	802	4.81	.07 [.07-.08]	.95	.94	.54-.82	-	-	.45	.46-.54	.85-.86	.85-.85
MBDS-R	BR	429	2.79	.06 [.05-.08]	.96	.95	.55-.84	-	-	.40	.46-.55	.86-.86	.85-.86
MBDS-R	PT	373	3.42	.08 [.07-.09]	.93	.92	.53-.81	-	-	.52	.46-.55	.86-.86	.85-.86
PHCS-B	BR/PT	802	13.40	.12 [.11-.14]	.96	.94	.60-.83	-	-	.62	.55-.56	.83-.83	.77-.78
PHCS-B (fitted)	BR/PT	802	5.80	.08 [.06-.10]	.99	.98	.71-.85	1, 3	-	.63	.59-.60	.81-.82	.76-.77
PHCS-B	BR	429	8.01	.13 [.11-.15]	.96	.94	.64-.82	-	-	.73	.54-.57	.82-.84	.78-.79
PHCS-B (fitted)	BR	429	4.33	.09 [.06-.10]	.99	.98	.66-.84	1, 3	-	.74	.58-.61	.80-.82	.76-.78
PHCS-B	PT	373	7.09	.13 [.11-.15]	.95	.92	.59-.82	-	-	.52	.53-.56	.82-.83	.76-.77
PHCS-B (fitted)	PT	373	3.89	.09 [.06-.10]	.99	.97	.70-.85	1, 3	-	.53	.57-.61	.80-.82	.74-.76
TFEQ-18	BR/PT	802	2.26	.04 [.03-.05]	.97	.96	.58-.94	-	-	.00*-.36	.43-.72	.86-.89	.70-.76
TFEQ-18	BR	429	1.82	.04 [.03-.05]	.96	.95	.47-.95	-	-	.00*-.39	.41-.75	.86-.90	.72-.75
TFEQ-18	PT	373	1.56	.04 [.03-,05]	.97	.96	.55-.90	-	-	.02-.34	.47-.70	.85-.89	.68-.78
WHOQoL-bref	BR/PT	802	5.56	.07 [.07-.08]	.92	.91	.51-.90	-	-	.28-.63	.34-.57	.78-.84	.68-.77
WHOQoL-bref	BR	429	3.31	.07 [.07-.08]	.92	.91	.44-.89	-	-	.24-.58	.29-.56	.74-.83	.68-.77
WHOQoL-bref	PT	373	3.30	.08 [.07-.09]	.93	.92	.47-.91	-	-	.30-.66	.37-.58	.78-.84	.68-.78

*Note*. We used adapted versions of instruments (cf. Instruments section). BSQ-8B = Body Shape Questionnaire (reduced version), MBDS-R = Male Body Dissatisfaction Scale (reduced version), PHCS = Perceived Health Competence Scale (bifactorial version), TFEQ-18 = Three-factor Eating Questionnaire (reduced version), WHOQoL-bref = World Health Organization Quality of Life Questionnaire-Short Form (refined version), fitted = instrument fitted for the study sample, Country: BR/PT = Brazil and Portugal, BR = Brazil, PT = Portugal, χ^2^/*df* = Chi-square by degrees of freedom, RMSEA = Root Mean Square Error of Approximation [CI = confidence interval of 90%], CFI = Comparative Fit Index, TLI = Tucker-Lewis Index, λ = factorial weight, EI = excluded items, e = items with correlation, *r*^2^ = coefficient of determination between factors, AVE = average variance extracted, CR = composite reliability, α = Cronbach's alpha.

* values <0,01.

It should also be noted, that the adequate fit of instruments was maintained when separated by country, corroborating with previous studies about adequate psychometric properties these measures in different contexts [33,43–45]. Moreover, it is important to clarify that some values presented in Table 1 for the χ^2^/*df* look to be out of adequacy. However, this index generally is inflated by the number of estimated measured parameters and also by the size of the sample. Thus, to support the adequacy of the evaluated structure, we use the RMSEA, which is a frequently used index and cited as one of the best quality indicators of adjustment of measurement models [40].

### Structural equation models

**Fig 1:**
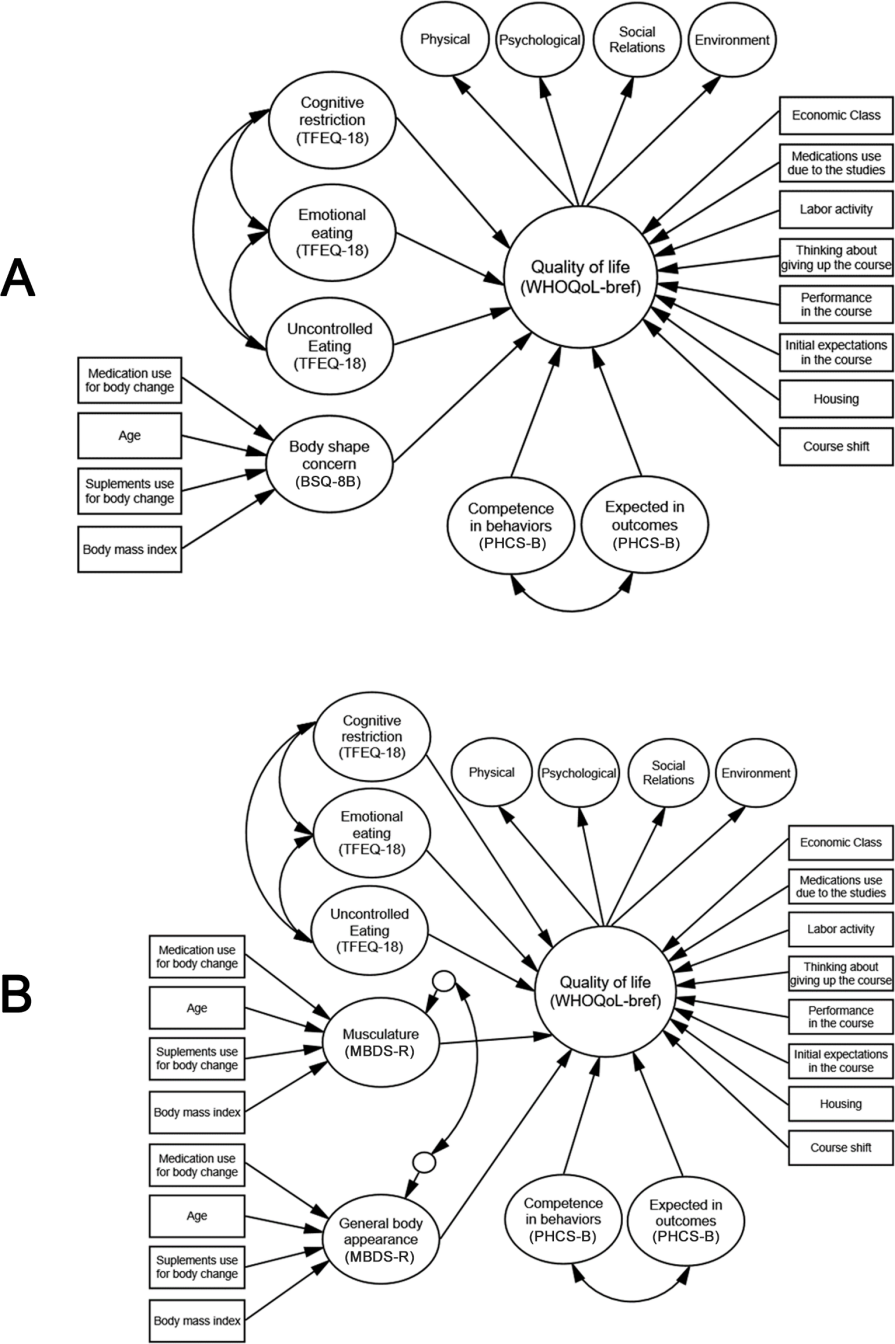
**Structural models with the hypothetical paths tested for female (A) and male (B) university students.** We used adapted versions of instruments (cf. Instruments section). BSQ-8B = Body Shape Questionnaire (reduced version), MBDS-R = Male Body Dissatisfaction Scale (reduced version), PHCS-B = Perceived Health Competence Scale (bifactorial version), TFEQ-18 = Three-factor Eating Questionnaire (reduced version), WHOQoL-bref = World Health Organization Quality of Life Questionnaire-Short Form (refined version). All the independent variables are correlated.

As mentioned previously, considering the differences between women and men regarding body image concept and the difference in instruments used, a separate structural model for each sex was tested. The paths that were tested in each model are presented in Fig 1.

In both models, the measured aspects (independent variables) of the body image (BSQ-8B = “body shape concern”, MBDS-R = “musculature”; “general body appearance”), of eating behavior (TFEQ-18 = “cognitive restriction”; “emotional eating”; “uncontrolled eating”) and of the perceived health competence (PHCS-B = “expectations of achieving the desired health results”; “competence in health behaviors”) were used as direct predictors in quality of life (WHOQoL-bref, dependent variable). The variables "age", "medication use for body change (1 = yes, 0 = no)", "food supplement use for body change (1 = yes, 0 = no)", and "BMI" were inserted in the aspects of the body image (i.e., in the BSQ-8B for the female model and in the MBDS-R domains for the male model). On the other hand, the variables "course shift (1 = diurnal, 0 = night)", "housing (1 = alone, 0 = family/friends)”, “initial expectation regarding the course (5 = much better, 4 = better, 3 = equal, 2 = worst, 1 = much worse)", "self-reported performance in the course (excellent = 4, good = 3, regular = 2, bad = 1)”, "thinking about drop out of the course (1 = yes, 0 = no)", "concomitant work activities to studies (1 = yes, 0 = no)", "medication use due to studies (1 = yes, 0 = no)”, and "economic class (4 = class A, 3 = class B, 2 = class C, 1 = classes D and E)” were inserted into the quality of life.

To evaluate the quality of the tested models, the recommendations suggested by Marôco [40] were adopted. Initially, the quality of the adjustment of the measurement model was evaluated through the indices χ^2^/*df*, RMSEA, CFI and TLI with their respective reference values (as presented in the section on psychometrical properties) and the WLSMV estimation method. Then, the significance of the hypothetically causal paths (β), calculated by the *z*-test at the critical ratios, were observed considering a significance level of 5%. The refinement of the models was performed by the stepwise method to identify the significant variables. The multicollinearity evaluation was performed through the Variance Inflation Factor (VIF) calculation, being the values of VIF>5 indicative of multicollinearity [40]. The models were constructed and analyzed in the software MPLUS version 7.2 (Muthén and Muthén, Los Angeles, USA, 2014).

## Results

At total of 2,857 of the students invited to participate in the study agreed to complete the questionnaires. However, 659 individuals did not correctly complete all items of all instruments and/or sociodemographic characteristics and therefore were not part of the study sample. Thus, 2,198 university students (women: *n* = 1,396 [Brazil: *n* = 722, Portugal: *n* = 674], men: *n* = 802 [Brazil: *n* = 429, Portugal: *n* = 373]) comprised the study sample. The average age of women was 20.8 ± 2.4 years (Brazil = 20.7 ± 2.2 years, Portugal = 21.0 ± 2.6 years) and of men was 21.3 ± 3.3 years (Brazil = 21.2 ± 3.2 years, 21.5 ± 3.5 years). Table 2 presents the characterization of the study sample.

**Table 2:** Characterization of the study sample.

	n (%)			
Characteristic	Female		Male	
	Brazil	Portugal	Brazil	Portugal
**Course area**				
Human	428 (59.3)	225 (33.4)	280 (65.3)	68 (18.2)
Exact	110 (15.2)	51 (7.6)	108 (25.2)	203 (54.4)
Health/Biological	184 (25.5)	398 (59.1)	41 (9.6)	102 (27.3)
**Year of the course**				
First	232 (32.1)	152 (22.6)	168 (39.2)	131 (35.1)
Second	205 (28.4)	106 (15.7)	96 (22.4)	114 (30.6)
Third	165 (22.9)	156 (23.2)	88 (20.5)	46 (12.3)
Fourth	80 (11.1)	193 (28.6)	46 (10.7)	53 (14.2)
Fifth	40 (5.5)	67 (9.9)	31 (7.2)	29 (7.8)
**Course shift**				
Day (morning, afternoon or full)	465 (64.4)	644 (95.5)	238 (55.5)	349 (93.6)
Night	257 (35.6)	30 (4.5)	191 (44.5)	24 (6.4)
**Housing**				
Alone	110 (15.2)	37 (5.5)	70 (16.3)	35 (9.4)
Family/friends	612 (84.8)	637 (94.5)	359 (83.7)	338 (90.6)
**Initial expectations about the course**				
Much better	108 (15.0)	82 (12.2)	61 (14.3)	46 (12.3)
Better	291 (40.3)	311 (46.1)	175 (40.8)	144 (38.6)
Equal	213 (29.5)	227 (33.7)	134 (31.2)	152 (40.8)
Worse	104 (14.4)	53 (7.9)	49 (11.4)	27 (7.2)
Much worse	6 (.8)	1 (.1)	10 (2.3)	4 (1.1)
**Self-reported performance in the course**				
Excellent	37 (5.1)	33 (4.9)	29 (6.8)	29 (7.8)
Good	455 (63.1)	417 (61.9)	218 (50.8)	171 (45.8)
Regular	211 (29.2)	217 (32.2)	151 (35.2)	154 (41.3)
Bad	19 (2.6)	7 (1.0)	31 (7.2)	19 (5.1)
**Thinking about giving up the course**				
Yes	384 (53.2)	193 (28.6)	193 (45.0)	123 (33.0)
No	338 (46.8)	481 (71.4)	236 (55.0)	250 (67.0)
**Labor activity concomitant to the studies**				
Yes	212 (29.4)	114 (16.9)	126 (29.4)	63 (16.9)
No	510 (70.6)	560 (83.1)	303 (70.6)	310 (83.1)
**Medications use due to studies**				
Yes	224 (31.0)	226 (33.5)	68 (15.9)	70 (18.8)
No	498 (69.0)	448 (66.5)	361 (84.1)	303 (81.2)
**Medications use for body change**				
Yes	85 (11.8)	92 (13.6)	72 (16.8)	41 (11.0)
No	637 (88.2)	582 (86.4)	357 (83.2)	332 (89.0)
**Food supplements use for body change**				
Yes	103 (14.3)	124 (18.4)	142 (33.1)	78 (20.9)
No	619 (85.7)	550 (81.6)	287 (66.9)	295 (79.1)
**Nutritional status**				
Underweight	43 (6.0)	52 (7.7)	8 (1.9)	13 (3.5)
Eutrophic	537 (74.4)	530 (78.6)	279 (65.0)	279 (74.8)
Overweight	113 (15.6)	78 (11.6)	107 (24.9)	68 (18.2)
Obesity	29 (4.0)	14 (2.1)	35 (8.2)	13 (3.5)
Economic class*				
A	197 (27.3)	47 (7.0)	137 (31.9)	37 (9.9)
B	377 (52.2)	259 (38.4)	208 (48.5)	159 (42.7)
C	145 (20.1)	322 (47.8)	82 (19.1)	149 (39.9)
D and E	3 (.4)	46 (6.8)	2 (.5)	28 (7.5)

*In Brazil.

The economic class was obtained using the average household income (Brazilian Criteria 2015) in Brazilian Reals (BRL) converted (exchange rate in October 2017) into American dollars (A = 6,628.15 USD; B = 2,238.91 USD; C = 687.26 USD; D and E = 243.79 USD). In Portugal, the classification was made using self-reported household income in Euros (EUR) converted (exchange rate in October 2017) into American dollars (A = > 2,968,89 USD; B = 1,781,34 USD; C = 1,187.56 USD; D and E = < 593.78 USD).

The structural models tested for each sex are presented in Table 3.

**Table 3:** Complete and refined structural models tested in Brazilian and Portuguese students of both sexes.

		Complete				Refined			
Model	Independent variable → Dependent variable	β	βs	SE	*p*	β	βs	SE	*p*
Female	Body Shape Concern (BSQ-8B) → Quality of Life (WHOQoL-bref)	-.237	-.284	.034	< .001*	-.120	-.151	.027	< .001*
	Cognitive Restriction (TFEQ-18) → Quality of Life (WHOQoL-bref)	.044	.049	.036	.175	-	-	-	-
	Emotional Eating (TFEQ-18) → Quality of Life (WHOQoL-bref)	-.071	-.084	.038	.028*	-.100	-.126	.031	< .001*
	Uncontrolled Eating (TFEQ-18) → Quality of Life (WHOQoL-bref)	-.043	-.042	.038	.275	-	-	-	-
	Expected Outcomes (PHCS-B) → Quality of Life (WHOQoL-bref)	.132	.118	.083	.158	-	-	-	-
	Competence in Behaviors (PHCS-B) → Quality of Life (WHOQoL-bref)	.545	.526	.084	< .001*	.454	.465	.026	< .001*
	Course shift → Quality of Life (WHOQoL-bref)	.154	.100	.029	.001*	.190	.128	.028	< .001*
	Housing → Quality of Life (WHOQoL-bref)	-.076	-.038	.026	.155	-	-	-	-
	Initial expectations in the course → Quality of Life (WHOQoL-bref)	.067	.095	.027	< .001*	.065	.095	.027	.001*
	Self-reported performance in the course → Quality of Life (WHOQoL-bref)	.241	.228	.026	< .001*	.240	.234	.026	< .001*
	Thinking about giving up the course → Quality of Life (WHOQoL-bref)	-.285	-.226	.027	< .001*	-.284	-.232	.027	< .001*
	Labor activity → Quality of Life (WHOQoL-bref)	-.078	-.053	.030	.077	-	-	-	-
	Medications use due to the studies → Quality of Life (WHOQoL-bref)	-.180	-.135	.026	< .001*	-.178	-.138	.027	< .001*
	Economic class → Quality of Life (WHOQOL-bref)	.031	.038	.026	.136	-	-	-	-
	Age → Body Shape Concern (BSQ-8B)	-.028	-.090	.030	.003*	-.025	-.079	.028	.005*
	Medications use for body change → Body Shape Concern (BSQ-8B)	.368	.165	.029	< .001*	.374	.164	.028	< .001*
	Food supplements use for body change → Body Shape Concern (BSQ-8B)	.177	.088	.028	.002*	.174	.085	.027	.002
	Body mass index → Body Shape Concern (BSQ-8B)	.085	.400	.022	< .001*	.088	.408	.021	< .001*
		*r*^2^ = .584, χ^2^/*df* = 3.206, RMSEA = .040 [CI 90% .039-.041], CFI = .899, TLI = .894				*r*^2^ = .539, χ^2^/*df* = 3.508, RMSEA = .042 [CI 90% .041-.044], CFI = .933, TLI = .929			
Male	Musculature (MBDS-R) → Quality of Life (WHOQoL-bref)	.006	.010	.048	.831	-	-	-	-
	General Body Appearance (MBDS-R) → Quality of Life (WHOQoL-bref)	-.139	-.162	.047	.001*	-.105	-.118	.034	< .001*
	Cognitive Restriction (TFEQ-18) → Quality of Life (WHOQoL-bref)	-.079	-.105	.040	.009*	-.101	-.128	.039	.001
	Emotional Eating (TFEQ-18) → Quality of Life (WHOQoL-bref)	-.071	-.093	.058	.112	-	-	-	-
	Uncontrolled Eating (TFEQ-18) → Quality of Life (WHOQoL-bref)	.045	.047	.053	.370	-	-	-	-
	Expected Outcomes (PHCS-B) → Quality of Life (WHOQoL-bref)	-.068	-.087	.067	.198	-	-	-	-
	Competence in Behaviors (PHCS-B) → Quality of Life (WHOQoL-bref)	.367	.448	.066	< .001*	.426	.526	.031	< .001*
	Course shift → Quality of Life (WHOQoL-bref)	.175	.134	.039	.001*	.185	.138	.035	< .001*
	Housing → Quality of Life (WHOQoL-bref)	-.042	-.024	.037	.512	-	-	-	-
	Initial expectations in the course → Quality of Life (WHOQoL-bref)	.101	.158	.036	< .001*	.109	.165	.036	< .001*
	Self-reported performance in the course → Quality of Life (WHOQoL-bref)	.111	.138	.035	< .001*	.111	.134	.035	< .001*
	Thinking about giving up the course → Quality of Life (WHOQoL-bref)	-.197	-.166	.036	< .001*	-.203	-.167	.036	< .001*
	Labor activity → Quality of Life (WHOQoL-bref)	.010	.008	.040	.850	-	-	-	-
	Medications use due to the studies → Quality of Life (WHOQoL-bref)	-.302	-.197	.035	< .001*	-.324	-.206	.035	< .001*
	Economic class → Quality of Life (WHOQoL-bref)	.034	.047	.036	.194	-	-	-	-
	Age → Musculature (MBDS-R)	-.017	-.059	.040	.140	-	-	-	-
	Medications use for body change → Musculature (MBDS-R)	.130	.048	.039	.218	-	-	-	-
	Food supplements use for body change → Musculature (MBDS-R)	.600	.285	.038	< .001*	-	-	-	-
	Body Mass Index → Musculature (MBDS-R)	-.012	-.047	.040	.238	-	-	-	-
	Age → General Body Appearance (MBDS-R)	-.015	-.075	.045	.096	-	-	-	-
	Medications use for body change → General Body Appearance (MBDS-R)	.033	.017	.042	.690	-	-	-	-
	Food supplements use for body change → General Body Appearance (MBDS-R)	.171	.113	.044	.010*	.181	.121	.038	.001*
	Body mass index → General Body Appearance (MBDS-R)	.018	.104	.038	.007*	.016	.093	.037	.012*
		*r*^2^ = .529, χ^2^/*df* = 1.822, RMSEA = .032 [CI 90% .030-.034], CFI = .906, TLI = .902				*r*^2^ = .491, χ^2^/*df* = 2.359, RMSEA = .041 [CI 90% .039-.044], CFI = .920, TLI = .914			

*Note*. The arrows refer to the direction of the paths that were used to build the model. We used adapted versions of instruments (cf. Instruments section). BSQ-8B = Body Shape Questionnaire (reduced version), MBDS-R = Male Body Dissatisfaction Scale (reduced version), PHCS = Perceived Health Competence Scale (bifactorial version), TFEQ-18 = Three-factor Eating Questionnaire (reduced version), WHOQoL-bref = World Health Organization Quality of Life Questionnaire-Short Form (refined version), β = estimate, βs = standardized estimate, SE = standard error, *r*^2^ = coefficient of determination, χ^2^/*df* = chi-square by degrees of freedom. RMSEA = Root Mean Square Error of Approximation [CI = confidence interval of 90%], CFI = Comparative Fit Index, TLI = Tucker-Lewis Index.

*p < .05

The complete models presented some non-significant paths and, therefore, were refined. In the female model, it was observed that the aspects "cognitive restriction", "uncontrolled eating" and "expectations of achieving desired health outcomes" and the variables "housing", "labor activity" and "economic class" did not contribute significantly to the quality of life of university students and thus were excluded. Furthermore, it was observed that item 11 of the WHOQoL-bref presented a high modification index (LM = 718.31), indicating a high correlation with the aspect "body shape concern (BSQ-8B)”, and in this way, we opted for exclusion of this item. After refinement, the female model presented only significant paths, adjustment adequate and explained the variance of 54%. Women who are less concerned with body shape, who do less emotional eating, perceive themselves competent in their behaviors to manage their own health, study during the day, have better expectations, perform well, who do not think about giving up on the course they attend, and who do not consume medications due the pressure of their studies have a better quality of life. It has also been observed that younger students who consume medication and food supplements for body change and higher BMI are more concerned with body shape.

In the male model, it was observed that the aspects "musculature", "emotional eating", "uncontrolled eating", "expectations of achieving desired health outcomes" and the variables "housing" and "labor activity" did not contribute significantly to the quality of life of university students and, therefore, were excluded. Still, "age" and "consumption of medications for body change" were not significant for the evaluation of "general body appearance", and were also excluded. Similar to the female model, item 11 of the WHOQoL-bref presented high indices of modification with the aspects "musculature" (LM = 172.80) and "general body appearance" (LM = 200.43) and were excluded. The refined model presented only significant paths, adjustment adequate and explained the variance of 49%. The students with lower body dissatisfaction, with less cognitive eating restriction, perceive themselves to be more competent in their own health behaviors, study during the day, have better expectations, who perform well and do not think about giving up of their course of study, and who do not consume medications due to studies, have better quality of life. It was also observed that male individuals who consume food supplements for body change and have a higher BMI presented greater dissatisfaction with general body appearance.

## Discussion

This study presented theoretical models with significant contribution to inherent aspects of body image, eating behavior and perceived health competence, and academic variables in the quality of life of Brazilian and Portuguese university students of both sexes. Some differences were observed between the sexes both with regard to eating behavior and significant variables in the evaluated aspects of body image.

In relation to the structural models tested, it was observed that different aspects impact the quality of life of female and male students. In the evaluation of the inherent aspects of body image, it was observed that the lower the concern with body shape by the women, and the less dissatisfaction with general body appearance by men, the better the quality of life of these individuals. Cox et al. [13] and Kolodziejczyk et al. [14] evaluated the relationship between some aspects of body image and quality of life in different samples and verified equally that the higher the concern/dissatisfaction of individuals with the body, the worse the quality of life. These studies corroborate our results and the need to raise public awareness of the value of physical and mental health, rather than targeting body patterns that are often unrealistic, unreachable and imposed by the society and widely publicized by the media.

Also, a significant contribution of the variables age, BMI, and consumption of medications and food supplements for body change as they relate to body image was observed. The relation between BMI and body image is commonly observed in the literature. Cox et al. [13] and Kolodziejczyk et al. [14] similarly found as in our study that individuals with higher BMI are more concerned with body shape, which impacts negatively on their quality of life. Younger women were also more susceptible to a greater concern with body shape in our study, and this outcome was also reported by Quick et al. [12] reinforcing the need for early intervention in this population. Regarding the use of medications and food supplements for body change, it is noted that students who consume these substances have greater concern/dissatisfaction with the body, which consequently impacts their quality of life. Hildebrandt et al. [15] and Yager and O'Dea [16] also identified a significant relationship between the use of medications and food supplements for body change and the body image in Australian and American individuals. These studies highlight the need for awareness regarding the use of these substances, as they can directly interfere with the physical health of individuals, as well as lead to the development of problems related to body dysmorphic disorders.

For the evaluated aspects of eating behavior, it was observed that the lower the level of emotional eating of women and the lower the cognitive eating restriction of men, the better the quality of life. Valladares et al. [46] evaluated the eating behavior of Chilean students and found that women presented higher scores of emotional eating. Poinhos, Oliveira, and Correia [47] also verified higher scores of emotional eating in Portuguese female university students, and pointed out the significant differences in the eating behavior of women and men. Besides, these authors identified that the "restriction" theme was commonly related to eating. Thus, it is noted that the measured aspects of eating behavior in the present study are relevant to the quality of life of university students. However, there are differences between the sexes. In our study, the eating based on emotion was directly tied to women. For men, it was found that eating restriction is an aspect inversely related to quality of life. We attribute this result to the fact that men generally do not usually restrict eating but when this occurs, it means altered eating behavior, which may have resulted in significant impact on quality of life. Thus, attention needs to be paid to strategies adopted by women and men regarding eating so that educational/preventive interventions aimed at changing eating behavior can help foster better life quality.

The significant relationship between perceived health competence and quality of life was found in the models tested in our study. Both female and male students found themselves competent to manage their own health, and this had a positive impact on the quality of life of this population. Salyer et al. [20] and Rueda and Perez-Garcia [4] studied the relationship between different aspects, including perceived health competence and quality of life in clinical samples. The results of both studies corroborate our findings that individuals who perceive themselves to be more competent in managing their own health had a better quality of life. Thus, this information reinforces the importance of encouraging individuals to identify any health problems, and find viable and effective solutions to manage their own health. Still, the evaluation of the perceived competence in health behaviors is important, because it aids in the identification of individuals who need additional support to deal with their own health statuses.

Regarding other characteristics that impacted significantly in the students' quality of life, the academic ones stand out. It was observed in both models (female and male) that university students that attend day classes, with better initial expectations in relation to the course, good self-reported performance in the course, who do not think about giving up the course, and do not consume medications due to their studies, had a better quality of life. The relation between studying during the day and a better quality of life may be based on the greater availability of time for exclusive dedication to academic activities. Night students usually work during the day and study at night, which can represent an overload and influence in the evaluation of quality of life (confidence interval 95% of the prevalence of students who reported working: men at night = 52.44–53.36%, men at day = 13.01–13.23%, women at night = 53.67–54.35%, women at day = 15.36–15.48%). Still, the relationship between better initial expectations and not considering giving up studies, and better quality of life, on the other, may be associated with the self-confidence, motivation, and positivity of these individuals in relation to careers chosen for the future.

The significant relation between good academic performance and better quality of life was also reported by Shareef et al. [23] in university students in Saudi Arabia. This result informs us that academic performance is an important characteristic in students' lives, and should be considered in the research/intervention protocols. Yet the relation between consume medications due the pressure of their studies and quality of life is seldom explored in the literature. However, some studies [48,49] highlighted that the prevalence of medication use by young university students is high, and one of the complaints related to their use is the routine of studies. Thus, the literature is in similar to our results suggesting that the pressures in the university can have a significant impact on students' quality of life. Therefore, the academic characteristics should be considered in investigative/intervention protocols.

In general, aspects of body image, eating behavior and perceived health competence, as well as academic characteristics, were significantly important in evaluating the quality of life of Brazilian and Portuguese university students. The significant relations among the study variables reveal, mainly, the need to create and implement educational programs aimed at promoting preventive health to promote an improved quality of life. In addition, the models evaluated for women and men presented variance explained of 54% and 49%, respectively, pointing out, the identification of relevant aspects to predicting the quality of life of university students.

Some limitations of our study should be mentioned. The first one refers to our cross-sectional study design that does not allow confirmation of the temporal cause and effect relation between the studied variables. However, cross-sectional studies may aid in the identification of the issues that should be considered in intervention studies. Second, in relation to the data that was collected in only in one institution in Brazil, and gathered in both countries using non-probabilistic methods, there are limitations in generalizing our results to this population. Third, the lack of transnational comparison (Brazil vs. Portugal) of the models found for women and men, which would require a larger sample in each country and samples paired between countries according to sociodemographic characteristics. Thus, in order to overcome the limitations of our work, we suggest other studies to verify the relationship between the studied characteristics in other samples.

## Supporting information

S1 Dataset Dataset for our study. (XLSX)
